# Applying the behavior change wheel to design de-implementation strategies to reduce low-value statin prescription in primary prevention of cardiovascular disease in primary care

**DOI:** 10.3389/fmed.2022.967887

**Published:** 2022-10-13

**Authors:** Alvaro Sanchez, Usue Elizondo-Alzola, Jose I. Pijoan, Marta M. Mediavilla, Susana Pablo, Rita Sainz de Rozas, Itxasne Lekue, Susana Gonzalez-Larragan, Marta Llarena, Olatz Larrañaga, Christian D. Helfrich, Gonzalo Grandes

**Affiliations:** ^1^Primary Care Research Unit of Bizkaia, Biocruces Bizkaia Health Research Institute, Basque Health Service-Osakidetza, Barakaldo, Spain; ^2^Clinical Epidemiology Unit, Biocruces Bizkaia Health Research Institute, Basque Health Service-Osakidetza, Barakaldo, Spain; ^3^Primary Care Pharmacy Unit, Ezkerraldea-Enkarterri-Cruces Integrated Health Organization, Basque Health Service-Osakidetza, Barakaldo, Spain; ^4^Department of Health Science Library, Biocruces Bizkaia Health Research Institute, Basque Health Service-Osakidetza, Barakaldo, Spain; ^5^VA Puget Sound Health Care System, Seattle-Denver Center of Innovation for Veteran-Centered and Value-Driven Care, Seattle, WA, United States; ^6^Department of Health Services, University of Washington School of Public Health, Seattle, WA, United States

**Keywords:** de-implementation, inappropriate prescribing, statin, cardiovascular disease, primary prevention, primary care

## Abstract

**Introduction:**

A substantial proportion of individuals with low cardiovascular risk receive inappropriate statin prescription for primary prevention of cardiovascular disease (CVD) instead of the evidence-based recommendations to promote healthy lifestyle behaviors. This study reports on the structured process performed to design targeted de-implementation strategies to reduce inappropriate prescription of statins and to increase healthy lifestyle promotion in low cardiovascular risk patients in Primary Care (PC).

**Methods:**

A formative study was conducted based on the Theoretical Domains Framework and the Behavior Change Wheel (BCW). It comprised semi-structured interviews with PC professionals to define the problem in behavioral terms; focus groups with Family Physicians and patients to identify the determinants (barriers and facilitators) of inappropriate statin prescription and of healthy lifestyle promotion practice; mapping of behavioral change interventions operationalized as de-implementation strategies for addressing identified determinants; and consensus techniques for prioritization of strategies based on perceived effectiveness, feasibility and acceptability.

**Results:**

Identified key determinants of statin prescription and healthy lifestyle promotion were: the lack of time and clinical inertia, external resources, patients' preferences and characteristics, limitation of available clinical tools and guidelines, social pressures, fears about negative consequences of not treating, and lack of skills and training of professionals. Fourteen potential de-implementation strategies were mapped to the identified determinants and the following were prioritized: 1) non-reflective decision assistance strategies based on reminders and decision support tools for helping clinical decision-making; 2) decision information strategies based on the principles of knowledge dissemination (e.g., corporative diffusion of evidence-based Clinical Practice Guidelines and Pathways for CVD primary prevention); 3) reflective decision-making restructuring strategies (i.e., audit and feedback provided along with intention formation interventions).

**Conclusions:**

This study supports the usefulness of the BCW to guide the design and development of de-implementation strategies targeting the determinants of clinicians' decision-making processes to favor the abandonment of low-value practices and the uptake of those recommended for CVD primary prevention in low-risk patients. Further research to evaluate the feasibility and effectiveness of selected strategies is warranted.

**Clinical trial registration:**

Sanchez A. De-implementation of Low-value Pharmacological Prescriptions (De-imFAR). ClinicalTrials.gov, Identifier: NCT04022850. Registered July 17, 2019. In: ClinicalTrials.gov. Bethesda (MD): U.S. National Library of Medicine (NLM). Available from: https://www.clinicaltrials.gov/ct2/show/NCT04022850.

## Introduction

De-implementation or abandonment of ineffective or low-value healthcare has gained great interest in recent decades, due, among other factors, to the growing empirical evidence of its high prevalence and its impact on patient safety, resource consumption and social inefficiency (1, 2). Accordingly, theoretical and empirical evidence-based knowledge about the key factors (barriers, facilitators, etc.) required for the design and application of de-implementation of low value care, is growing rapidly (2–4).

In light of current understanding of how to tackle targeted implementation and de-implementation endeavors, several fundamental aspects can be concluded. Firstly, factors driving the implementation of both evidence-based and inappropriate interventions in the clinical setting are multi-level, complex, and context specific (2). Therefore, a wide range of factors related to the intervention or practice to be de-implemented, the agents involved in this practice (e.g., healthcare professionals, patients) and other inner and outer context factors, should be carefully accounted for. Secondly, in order to be able to change a certain clinical practice, involved agents and stakeholders should be engaged in the process of identifying the practice determinants, in the design of focused interventions and implementation strategies and in the planning of their assessment (5, 6). Finally, the intervention design should be performed following a formal analysis of the target behavior and its mechanisms of action, and guided by models or theories covering the whole range of potential behavior influences or determinants at stake (7, 8). To this end, Behavioral Science and behavior change theories and frameworks for the development or planning of interventions can aid in better identifying and understanding the multi-level mechanisms that altogether influence clinical behavior, as well as in the selection of focused, effective techniques to promote behavior change of healthcare professionals (9, 10). Some examples of such theories or frameworks for the development or planning of interventions are the PRECEDE-PROCEED model (11), Implementation Mapping (12), or the Behavior Change Wheel (BCW) (13). Despite the wealth of recent scientific literature on the development of intervention or implementation strategies to facilitate the uptake of innovative or evidence-based practices, the application of behavioral science theory for the development of de-implementation interventions is scarce (14–16).

The DE-imFAR (from the Spanish for DE-implementation of low-value pharmacological prescriptions) study aims to carry out a structured, evidence-based and theory-informed process involving the main stakeholders (healthcare managers, professionals, patients, and researchers) for the design, deployment and evaluation of targeted de-implementation strategies for reducing low-value pharmacological prescribing (17). Specifically, the selected low-value practice is the prescription of statins in primary prevention of cardiovascular disease (CVD) in patients with low cardiovascular risk (CVR) (i.e., <10% CVR according to the REGICOR equation). Based on the evidence (18) and clinical practice guidelines (19–21), it is recommended not to start statin therapy in this population, with promotion of healthy lifestyles (i.e., healthy diet, physical activity and smoking cessation) being the recommended intervention instead.

Despite these recommendations, over the last years the consumption of statins in the Basque Country has increased substantially due, in part, to a CVD prevention approach excessively focused on the control of lipid levels and the use of medications. In this regard, the results from a descriptive observational study (as part of the DE-imFAR study) with data from electronic health records (EHR) on the inappropriate prescription rate of statins in patients aged 40–75 years with no history of CVD, with moderately cholesterol but with a CVR <5% (REGICOR) showed an incidence of new inappropriate prescriptions of 10.5 per 100,000 people/year (22). Furthermore, over 60% of the EHRs of these patients with inappropriate prescription of statins did not have a record of having been given advice on physical activity or a healthy diet. Likewise, 49% did not receive preventive recommendation on smoking cessation (22).

This paper reports on Phase I of the DE-imFAR study. Its main aim is to conduct a formative research in the specific context of the Basque Health Service-Osakidetza to: i) understand the problem of low-value statin prescription in primary prevention of CVD and define it in behavioral terms; ii) identify the main determinants of this clinical practice (e.g., at personal, inter-personal, organizational, social level) that must be addressed to change this behavior, and iii) map potential de-implementation strategies; and iv) prioritize mapped strategies based on their perceived effectiveness, feasibility and acceptability.

## Materials and methods

### Design

A Phase I formative study applying systematic, comprehensive and evidence-based frameworks, such as the Theoretical Domains Framework (TDF) (23, 24) and the BCW (13, 25) for the collaborative design and development of de-implementation strategies to favor the abandonment of low-value pharmacological prescribing of statins in primary prevention of CVD. The DE-imFAR study protocol was reviewed and approved by the Basque Country Clinical Research Ethics Committee (Reference: PI2019102, approved on 10 April 2019) and was registered in the U.S. NLM ClinicalTrials.gov database (ClinicalTrials.gov Identifier NCT04022850, 17 July 2019).

A working group, which was composed of experts in the design of implementation strategies, methodologists, pharmacists, qualitative researchers, clinicians and health service managers, used the TDF and BCW to identify, select, adapt and define possible behavioral change interventions operationalized as de-implementation strategies to address the prioritized determinants of inappropriate statin prescription in CVD primary prevention. This process involved eight steps grouped into the following three stages:

#### 1st stage-understand the behavior: Step 1) define the problem in behavioral terms; step 2) select the target behaviors; step 3) specify the target behaviors; step 4) identify what needs to change

We conducted a set of five semi-structured interviews with a sample of Family Physicians (FPs) (*n* = 3) and Practice Nurses (*n* = 2) with recognized expertise and experience in CVD prevention in order to identify the overall behavioral scenario and break down the chain of behaviors and concomitant non-behavioral (e.g., contextual) factors (step 1). The interview script was centered on determining how physicians address and manage the clinical encounters related to CVD prevention, and what the main steps taken are. Three members of the working group independently reviewed the recordings of the interviews, and identified and proposed a set of possible target behaviors. Subsequently, using matrices and exercises proposed by the BCW (25), the working group proceeded to vote and discuss until agreement in order to select (step 2) and to specify (step 3) the final target behaviors most likely to lead to the desired behavior change.

In order to explore the practice determinants (barriers and facilitator) of the selected target behaviors related to inappropriate statin prescription and healthy lifestyle promotion actions, a qualitative study comprising focus groups with FPs was performed (step 4).

Since the DE-imFAR study was carried out in two of the 13 Integrated Healthcare Organizations (IHO) of Osakidetza, a convenience sampling strategy for the recruitment of the healthcare professionals was used. In short, emails were sent to all the FPs from the Ezkerraldea-Enkarterri-Cruces (*n* = 83) and Barakaldo-Sestao (*n* = 123) IHOs with a brief explanation of the project and the invitation to participate. Out of the total number contacted, it was possible to recruit 21 FPs. Four focus groups of about 90 min of duration were conducted, two for each IHO, with between four to seven attendees in each group.

The groups were led by two researchers with experience in qualitative research methods, as well as knowledge of the clinical field. The focus groups were audio recorded and transcribed verbatim. Informed consent from all participants was obtained prior to any research procedure. The script of the focus groups were developed to explore in-depth potential determinants with questions covering each of the TDF dimensions (24). An inductive analysis based on grounded theory (26) was adopted to favor the emergence of a theory on the functioning of inappropriate statin prescription based on the words and phrases of the professionals. To facilitate the analysis, a coding scheme regarding the TDF dimensions and their relative constructs was developed. Two researchers independently reviewed and coded the transcripts and iteratively discussed possible discrepancies until reaching a consensus.

In addition, and with a twofold goal of firstly, ascertaining patients' experience regarding the clinical practice of statin prescription; and secondly, of triangulating physicians discourse, a focus group with patients was also conducted. To do so, participating professionals were requested to provide a list of patients “at low cardiovascular risk, in treatment with statins,” as well as for authorization to contact them. Eleven identified patients were contacted by telephone explaining the general objective of the study and the specific purpose of the discussion group and were invited to participate. Finally, a discussion group made up of six patients who agreed to participate was held. Informed consent from all patients was obtained prior the focus groups commencement. The following aspects were explored: how the pharmacological treatment was started; whether it was a decision made in conjunction with the FP; how they were informed; what factors could determine this action (preference or health problem, and at patient, professional, health center level), patient comfort with treatment, and so on.

#### 2nd stage-identify intervention options: Step 5) select intervention functions; step 6) select the specific behavior change techniques

The goal at this stage was to identify the Behavior Change Techniques (BCTs) for each of the agreed determinants of selected target behaviors. Two researchers proceeded first to group each identified barriers and facilitators into their respective TDF domain. Subsequently, they described identified determinants in the form of “what needs to change” and linked them to the intervention functions guided by the BCW instructions and suggestions. Then, all potential policy categories were identified. Lastly, potential BCTs most likely to produce a change were selected for each determinant using the process established by the BCW (25).

#### 3rd stage-identify implementation procedures: Step 7) select strategies and intervention techniques; step 8) select the mode of execution of the intervention

Final definition, packaging and selection of previously identified de-implementation strategies were carried out through a participatory consensus process in the form of round meetings involving the working group as representatives of the main stakeholders. In short, the working group first grouped and logically ordered all related mapped BCTs (i.e., those hypothesized to address the same determinant or several determinants at a time). Then and guided by examples of de-implementation interventions within the literature and by the experience of several team members in the design of implementation strategies, the working group decided upon a clear layout of the techniques to be applied (i.e., the actual content of the interventions, their possible formats and modes of execution) for each of the potential interventions identified through this structured mapping process.

#### Priorization of identified de-implementation strategies

Finally, in order to prioritize the de-implementation strategies derived from the conducted mapping, a poll process using the LimeSurvey platform involving FPs who collaborated in the focus groups was carried out. Specifically, they assessed the potential effectiveness, acceptability and feasibility of each identified strategy. The prioritization analysis, taking into account the ordinal nature of the measurement scale, was carried out by counting the number (proportion) of observations in each value of the assessed variables. Those considered potentially effective while highly acceptable and feasible for enacting behavior change were prioritized as the final set of specific strategies, to be contained in at least one broad de-implementation strategy seeking to reduce low-value pharmacological prescribing in the primary prevention of CVD.

## Results

### 1st stage-understand the behavior

#### Steps 1, 2, and 3. Define the problem in behavioral terms, select the target behaviors, and specify the target behaviors

Firstly, derived from the five semi-structures interviews with FPs and Nurses conducted in step 1, the working group defined the target behavior as: “*Reduce the prescription of statins in the context of primary prevention of CVD in low-risk patients (REGICOR* <*5%) and favor the adoption and implementation of the recommended intervention, the promotion of healthy lifestyles (regular physical activity, healthy diet and giving up smoking) at any opportunistic or programmed health center visit for screening or addressing CVD risk factors”* (Supplementary Table S1).

Afterwards, in steps 2 and 3, this target behavior was broken down into the chain of behaviors involved and the concomitant precipitating factors (Supplementary Table S2). Three precipitating factors for the practice of primary prevention of CVD were identified: i) alarm systems integrated within the EHR prompting the fulfillment of the Preventive Activities Program (PAPPs); ii) the presence of high cholesterol levels in a blood test result; or, iii) the presence of a prescription initiated or suggested by another professional (specialist or private). Regarding the preventive action behaviors by FPs and Practice Nurses, seven main steps were identified, ranging from the initial general approach for CVD primary prevention focused on CVD risk and the cholesterol level to the enactment of the decided treatment or intervention, the options being the prescription of a statin, the delivery of a healthy lifestyle promotion intervention, or both. The following specific behavior was prioritized by the working group and described according to who needs to do what, when, where, how often, how and with whom, as that most likely to bring about change: “*The FP considers options and makes the clinical decision on intervention/treatment to be provided, based on the result of the CVD risk estimation, on knowledge and heuristics in relation to the recommended practice, their attitudes, expectations and abilities, and other contextual factors (time, work overload, organizational norms, decisional fatigue, etc.).”*

#### Step 4. Identify what needs to change

Numerous determinants, facilitators of the inappropriate statin prescription and barriers toward healthy lifestyle promotion emerged from the focus groups with healthcare professionals. Determinants were identified from the quotes guided by a pre-specified coding. Though professionals' discourse tended to saturation, we do not have explicit confirmation of having reached saturation of data with the four groups. Table 1 displays some examples of quotes classified by the domains of the TDF. Except from one TDF dimension, Optimism, all the rest of the dimensions were covered in the FPs' discourses (see Table 1 for extracted quotes):

**Table 1:** Quotes extracted from FPs discussion groups by theoretical domains framework determinant dimensions.

**TDF dimension**	**Extracted quotes**
Knowledge	“*When you called me, what struck me was that I don't see so many people who should not take statins and are taking them.” (K_Q1)*
	“*Then also, the issue of the reliability of the guidelines is an issue... the sensitivity and specificity you have when making a decision... the issue of cholesterol is quite controversial.” (K_Q2)*
	“*Cholesterol levels have been very variable, and we didn't know if it was necessary to treat this in primary or secondary prevention, but then it became clear that it was in secondary, not in primary, that diabetics are in secondary, and if they're not... there we've also had a bit of trouble and so that could also be the cause of this prescription” (K_Q3)*
	“*I think that we have to be clear about that at least, that there's no evidence for giving statins, unless there's a family history, yes.” (K_Q4)*
	“*We are seeing that there are other added risk factors, there are diseases that we are seeing that have a greater risk of having that disease, rheumatism for example, but some other things aren't. In the analysis that you have made of Osakidetza, this might be there or not, but you probably haven't been able to see if they have a family history of sudden death, you cannot see if in addition to this they have other diseases that have to do with greater risk, which are being seen today. We don't see many of these.”(K_Q5)*
Skills	“*..For us it is also easier to prescribe a pill… It's simple, I ask you to take a test in two months, and ask ‘Is everything okay? Does anything hurt? See you next year’ and, that's it, it was a test and two appointments.” (Sk_Q1)*
	“*We have a training deficit in terms of the prescription of physical exercise and the prescription of nutrition in general and if you have some training it is because you have asked for it, because you have read about it, because you have shown interest. I believe that the way we are working, it is very complicated in the appointment with the patient, with the time we have and all the things we have to do…” (Sk_Q2)*
Beliefs about capabilities	“*It is much harder to change the habits of someone who comes to have their cholesterol tested if they are about 40 or 45, with settled habits that are difficult to change… that's harder than, ‘Give me a pill and I am going to do it quickly’, and I have peace of mind.” (Cap_Q1)*
	“*Walking progressively without getting tired, that works for everyone. I am not ready to prescribe physical activity. I think we can, but it is not effective.” (Cap_Q2)*
	“*This age group is people who are working and do not come to consult you except when they are sick for some reason, so they often pass under the radar. You ask them for a test, and their cholesterol is skyrocketing, but you don't get them to come to a consultation to see where they are failing, to be able to treat changes in habits... it is difficult to make them come to the health center, and it is also difficult to get them to make the changes... I think that there is a lot we don't see.” (Cap_Q3)*
Beliefs about	“*And the decision is always going to be, just in case, I'm going to give it to them. And then you also defend yourself just in case.” (Con_Q1)*
consequences	“*Also, in the real world, statins are a spectacular, very effective drug. I have 270 cholesterol, I go on a diet or exercise and I get down to 240 and that's that. However, if I take the pill, after 3 months I am at 200” (Con_Q2)*
	“*On the one hand we have the problem on both sides, we who find it more work and have a reward in the medium to long term in terms of results, and on the other hand what the user wants is immediacy now. They've come to ask us to solve it now.” (Con_Q3)*
	“*Patients also hear that statins are bad, that they can cause diabetes and brain hemorrhages... some stop taking them because they have heard that it can cause some problems, or there have been people who for muscular reasons have had to stop taking them and take others... there was one statin that came out and they had to withdraw it from the market... all of these are little things... but, well…” (Con_Q4)*
Motivation, goals, intent	“*My experience is that maybe you have been saying to the patient for 2 or 3 years, ‘You have to take exercise, go for a walk’… and they always look for an excuse, ‘I can’t because of my work…', so in the end you say, ‘Well, leave it then’ and you give up.” (M_Q1)*
	“*In the end it depends on the conviction that you have, if you are more convinced, you will dedicate more time. Personal conviction and what you want.”(M_Q2)*
Memory, attention, decision making	“*We doctors are inert by definition. Clinical and therapeutic inertia is part of our makeup. We are very inert, whether to prescribe or to stop prescribing.”(MAD_Q1)*
	“*Often, when you are not sure, the most normal thing that we doctors learn is to see something and prescribe, as that it is the fastest thing we have…. so we don't have to explain… it's easier to give medicine than to explain.” (MAD_Q2)*
	“*You are seeing patient 141, you are already tired, and someone has made an appointment for you to give them statins, they tell you that if something happens to them you will be responsible... And on top of it all, at that time of day you have low blood sugar... I ask you how you would manage that situation.” (MAD_Q3)*
	“*...the matter of the asterisk, and what happens when we see one... just today someone came to me with cardiovascular risk of 3 or 4, and had an LDL that was almost 190. This was a young woman of 40, with low cardiovascular risk, and she asked me if she had to take something for it.” (MAD_Q4)*
	“*And one thing, they should take away the asterisks, as we spend a lot of time explaining asterisks when we shouldn't have to.” (MAD_Q5)*
Environmental context, resources,	“*Sometimes, most of the time, we don't have enough time, and the time factor is important for everyone I think, to tell them, to try to convince them.” (E_Q1)*
constraints	“*...I think that the pressure of attending patients may have too much influence on the matter of prescription.” (E_Q2)*
	“*The Regicor does not mean you stop being a doctor, you have to continue being a doctor, just like we use the stethoscope as a tool. And the problem of the risk scale is good for the population, it is very good for population risks, but not for individuals, they weren't designed for that.” (E_Q3)*
	“*Well, that allows me to put if the patient is in primary or secondary prevention, if they have anxiety or not, are stressed or not... that allows me to modulate those risk modifiers, and gives me peace of mind in both senses. This patient doesn't need statins, I'm sure, and that one does need statins, certainly.” (E_Q4)*
	“*My nurse does it very well. I am very lucky, she is a highly trained woman who does it very well. So I delegate some things to her. But unfortunately, nowadays she is not always there, and not all nurses are trained...” (E_Q5)*
	“*… But it has to be at another level, multidisciplinary, health policies, health policies, lifestyle, which do not necessarily have to be based at the health center. It should also be involved but should not be the greatest weight and we should invest more in health policies especially in these types of people, the population base with least risk but who in the end are the ones that we can really prevent getting ill.” (E_Q6)*
	“*This age group includes people who are working and do not come to consult you except when they are sick for some reason, so they often pass under the radar. You ask them for a test, and their cholesterol is skyrocketing, but you don't attract them to a consultation to see where they are failing, to be able to treat changes in habits. That is the problem that I think we have in this age group. With older people who come to the health center more often, it's much easier. But with people who are at work... it is difficult to make them come to the health center, and it is also difficult to get them to make the changes... I think that there is a lot we don't see.” (E_Q7)*
	“*It is very difficult to get hold of them and to continue to call them in to make them get tests, like cholesterol, as they don't think much about prevention, because nothing hurts, and on top of that you restrict them a little, and in their life it is difficult for them to make those changes of habits so they don't come.” (E_Q8)*
Social and professional role and	“*I've had the experience of stopping a patient's statins, and the endocrinologist asked them what the family practitioner thought they were doing, taking them off statins... and then in the end the endocrinologist or the cardiologist put them back on them.” (Rol_Q1)*
identity	“*You see that a patient who has been to the... endocrinologist or... a patient who is seen in oncology, then comes to us in a state because they tell them that the doctor has to lower their cholesterol. These colleagues have a completely different view from ours, that this is a disease, and it can be important, except for very high numbers, which is a separate issue. The cardiologist who sees patients every day with heart attacks and things like that is much more likely to prescribe statins than we are, who see that much less.” (Rol_Q2)*
	“*This work is a bit beyond our usual work, but it should be a bit, it should direct us to giving a good prescription for physical education, where we can do this, or where there can be a good health provider who works in this way.” (Rol_Q3)*
Social influences	“*Cholesterol doesn't hurt, but it is so well-known that people are terribly afraid of it. On the other hand, they are not afraid of weighing 100 kilos, or smoking, or not exercising, but cholesterol is something objective… ”(SI_Q1)*
	*11Maybe the message of the media has a lot of influence, maybe we should try to change it, so that people become more aware of what cardiovascular risk means, as they're not aware. I think that's where we spend most time, explaining it to them.” (SI_Q2)*
	“*I believe that, on this issue, unlike other health issues, people come with a very preconceived idea, because there is pressure. In fact, when people do some tests, the first thing they ask you when they come for the results, is how high their cholesterol is.” (SI_Q3)*
	“*But I am referring to the advertising in which exercise, healthy food is being promoted more… that is what needs to be promoted. In the past, people didn't know much about exercise, but now they are a little more aware. Another thing is to get them to do it on a regular basis. That is what is difficult for the patients.” (SI_Q4)*
	“*For the patient, when you explain these dietary hygiene measures, it's like you aren't telling them anything... ‘What did the doctor tell you? Nothing, the usual...’ So it has little weight and little value for them, it's like not telling them anything. However, if you give them a pill and send them to have tests, that's different.” (SI_Q5)*
	“*Sorry, I have to go now. I signed up for a congress to prescribe exercise, and they didn't accept me. I was amazed. The reply from the person in the department where I applied was: “That is not a primary medicine matter.” I was amazed. To cap it all, I was the first at that time.” (SI_Q6)*
	“*We travel thanks to the pharmaceutical companies and we go to congresses thanks to the pharmaceutical companies and inadvertently there is always some contact in some way because they have given us training, which our company didn't do...” (SI_Q7)*
	“*I suppose these are the questions that (patients) often keep asking themselves, due to ignorance of the professionals, due to pressure from pharmaceutical companies, the media... and they think that if you don't take it you will have a heart attack, sure.” (SI_Q8)*
	“*…. there is a lot of obesity, people eat very badly... you tell them, eat fish. Maybe fish is the most expensive thing there is, maybe that person cannot afford it... there are many factors at play.” (SI_Q9)*
	*I believe that the socioeconomic and cultural level of the patients is very important because it's the people who have a lower cultural and socioeconomic level who are the ones we should invest in more, though it is harder for us, we know that we have to try harder. (SI_Q10)*
Emotion	“*Then too, the issue of the reliability of the guidelines is an issue... the sensitivity and specificity you have when making a decision... the issue of cholesterol is quite controversial.” (Em_Q1)*
	“*Perhaps I should also add, to all these causes which are variable, that at the beginning it was necessary to treat cholesterol no matter what. So perhaps we also have that inertia internalized, followed by all the other factors. The cholesterol figures have been very variable, we did not know if it was necessary to treat it in primary or secondary prevention, and then it was clarified and it was in secondary, not in primary, diabetics are secondary, if they are not... there we have also had a bit of a mess so that could also be the cause of this prescription. (Em_Q2)*
	“*I think it also affects you a bit, that little voice in the head that we all have, that maybe you still find a cholesterol level of 300 with low cardiovascular risk and you say, uff, they have 300, the risk is 2 and a half... and even though you yourself have explained to the patient and others, that also influences things, I mean, what if... and then there's what [name of healthcare professional] said about the penetration on the subject of cholesterol in all areas, which makes you always think about it, and say, what if I don't treat them?” (Em_Q3)*
	*It is much harder to change the habits of someone who comes to check their cholesterol when they are 40 or 45, when they are set in their ways, which are difficult to change... it is harder than ‘Give me a pill so that I will do it quickly and have peace of mind’. (Em_Q4)*
	*The ease, it is very easy to prescribe and it is also easily observable with the figures, that's it,... You feel good and the patient too (Em_Q5)*
	“*If you can get a patient to lose those kilos and on top of that stop smoking, there is no tool to measure it, but that's a great satisfaction.” (Em_Q6)*
Behavioral regulation	*I always comment on a lack of quality in the health center… and I still see that we do not stop and think, that there is no culture of quality evaluation, of demanding minimum standards and it seems to me that it's the most serious thing wrong with the public services. (BR_Q1)*
	*I think [data] is useful and we are all open to using it. When you are under this healthcare pressure, you are not aware of the way you are working day to day, if you see 30 patients a day, you do not remember if you have prescribed 2 statins or... I do not see it as intrusive, I see it as data, it helps me, it is a reflection. (BR_Q2)*
	…* Motivation is what drives everything, being aware of it. And for practical purposes I would ask the company for a tool…. I often want to see how my patients are doing, how many diabetics, under what conditions, but I can't. Before, we asked for this information and they gave it to us, but after a while you had to ask again... We shouldn't have to ask for it, we should be able to access it... to monitor yourself and do self-evaluation and then that's what would really change, if the company asks me, ‘Hey what are you doing?’… (BR_Q3)*
	*What was really useful for me in the center is to make small resolutions to make small changes that you are willing to make and that you feel capable of making, and once you have done them it is much better to keep them and then make a few more and If you have not been able to do them, you have to work on why not, if it was too excessive, if you think you can do a little less, if you can change it and solve it. (BR_Q4)*
Reinforcement	*But [name of healthcare professional], if you don't comply, what happens? And if you comply, what happens? Nothing, neither positive nor negative incentives, so… (Re_Q1)*
	*If you do it really well, and I do it really badly, they pay us the same, so… it doesn't matter. (Re_2)*

##### Knowledge

Participants felt that lack of awareness of the problem, doubts, clinical guidelines being out of date, and lack of consensus on or variability of recommendations, are main facilitators for an inappropriate prescription (see quotes K_Q1-Q3; Table 1). They believed that clearer evidence and getting a broader vision considering further risk factors would help to prescribe properly (K_Q4, Q5).

##### Skills

Differential required skills of alternative behaviors, statin prescription versus healthy lifestyle promotion, due to their perceived or experienced ease/difficulty seem to be, on the one hand, a facilitator of an inappropriate prescription, and on the other hand, a barrier to the recommended practice to be provided, especially regarding the prescription of physical activity (Sk_Q1,Q2).

##### Beliefs about capabilities

The main determinant related to capabilities is the low perceived confidence in prescribing healthy lifestyles, a clinical practice considered difficult in itself as compared to prescribing a statin (Cap_Q1, Q2). This problem is augmented by the difficulties faced by professionals to tackle healthy lifestyle promotion actions as a means of preventing CVD in low-risk patients, who are not usually frequent attenders (Cap_Q3).

##### Beliefs about consequences

The fear of negative consequences of not treating seemed to be a powerful driver of inappropriate prescribing (Con_Q1). This “defensive medicine” was also enhanced by the perceived effectiveness of statins in decreasing cholesterol levels (Con_Q2). Obtaining such a positive clinical result in the short term contrasted with the long term (and somewhat unperceived) benefits of healthy lifestyle promotion actions (Con_Q3). The adverse effects associated with statins seemed to be a potential barrier to statin prescription (Con_Q4).

##### Motivation, goals, and intent

The abovementioned scarcity of positive expected results from healthy lifestyle promotion actions has derived in a low motivation of professionals (M_Q1). Actual intention in the form of action plans or goals, both for not prescribing statins and for providing healthy lifestyle promotion interventions, is seen as a necessary condition to endorse guideline-concordant CVD primary prevention efforts (M_Q2).

##### Memory, attention, and decision-making

A repeated theme in physicians' discourse is the influence of clinical inertia in decision-making favored by contextual factors such as lack of time and heavy workload (MAD_Q1, Q2). Pharmacological prescription is perceived to require less cognitive effort in a saturated clinical practice that leads to decisional fatigue. A defensive medicine mindset is always present when deciding upon treatments (MAD_Q3). Physicians also requested the removal of asterisks in patients' blood test results (i.e., an asterisk is placed alongside cholesterol level when value is greater than or equal to 200 mg/dl) as this visual stimulus induces patients' concerns regarding cholesterol levels (MAD_Q4, Q5). Such markers incite cholesterol-control-focused clinical actions.

##### Environmental context, resources, and constraints

As previously commented, lack of time and the heavy workload experienced in Primary Care are the main obstacles for prevention efforts (E_Q1, Q2). Physicians also perceived that tools within the EHR are useful but somewhat limited for estimating cardiovascular risk, for reminding and fomenting guideline-concordant CVD primary prevention practice, and for restricting inappropriate statin prescribing (E_Q3, Q4). Teaming up with an involved Practice Nurse in order to share prevention efforts facilitated adequate healthy lifestyle promotion actions in primary prevention of CVD (E_Q5). Lack of external resources inside and outside the clinical setting (i.e., allied healthcare professionals, community resources, etc.) limits the reach of prevention efforts, especially in low-risk young adults, as a non-frequent-user population (E_Q6-Q8).

##### Social and professional role and identity

Lack of coherence in prescription criteria among the different healthcare professionals (i.e., cardiologists, neurologists and interns in addition to FPs) who attend the same patients dilutes responsible clinical practice (Rol_Q1, Q2). Uncertainties regarding limits in responsibility with respect to healthy lifestyle prescribing and fear of questioning each other's clinical decisions help to maintain inappropriate treatments (Rol_Q3).

##### Social influences

Patients' lack of awareness together with a perception of low susceptibility and vulnerability regarding cardiovascular risk hamper physicians' primary prevention efforts (SI_Q1, Q2). In contrast, due to the importance given by the media and probably fueled by the pharmaceutical industry, cholesterol is “the bad guy” everybody is worried about and needs to be addressed (SI_Q3). Another ambivalence occurs with healthy lifestyles. On the one hand, the population seems to be more conscious about the overall benefits of healthy behavior. But on the other hand, patients seem to have become so used to the message about the need to change to healthy lifestyles that some prefer to take a “magic” drug in the belief that there is no need to change habits (SI_Q4, Q5). In fact, neither the internal context in the health system which does not prioritize healthy lifestyle promotion practice, nor the external context at societal level influenced by media messages and the economic interests of the pharmaceutical industry targeting cholesterol reduction exclusively, are conducive to good CVD primary prevention practice (SI_Q6-Q8). Professionals also perceive that in certain sectors of the population, such as those with lower socio-economic status, the promotion of healthy lifestyle, although being the recommended practice, is very difficult to implement (SI_Q9, Q10).

##### Emotion

Mixed emotions were reported by physicians who mainly favor inappropriate prescribing. Professionals must make decisions in an emotional climate marked by uncertainty due to the variability of recommendations and limitations of the Clinical Practice Guidelines (CPG) and fear of consequences of not treating (Em_Q1-Q3). The feeling of pleasing the patient coupled with peace of mind after prescribing statins and obtaining “positive” cholesterol results are factors that seem to weigh substantially on decision making (Em_Q4, Q5). In contrast, positive emotions associated with successful healthy lifestyle changes seen in patients are the only emotional asset that favors continuing the work of promoting healthy lifestyles (Em_Q6).

##### Behavioral regulation

Professionals complained of a poor quality assessment culture in the healthcare system and of lack of standards and indicators established by the organization to anchor and guide clinical performance (BR_Q1). Data are needed to be able to reflect on performance and to be able to set goals, monitor progress and provide useful feedback, and the lack of access to such data prevents reflection and the establishment of objectives, both of which are seen as necessary to correct the problem of inadequacy in drug prescription (BR_Q2-Q4).

##### Reinforcement

In addition to the mentioned above in relation to objectives and performance indicators, the results of the evaluations of indicators carried out by the organization do not translate into incentives for professionals, which generates demotivation among those professionals willing to do things right (Re_Q1,Q2).

In addition, we conducted one focus group with six patients in order to triangulate professionals' discourse. We must highlight that the majority of the participants indicated a lack of explanation of the prescribed treatment and their desire to be more involved in the treatment decision. Moreover, they believed that family health history has a lot of weight in the decision and they are concerned about it. They reported that only some professionals recommended healthy lifestyles with or without prescription of statins. When we asked about their preferences for doing physical activity or taking a cholesterol-lowering drug in a context of low CVR, different points of view arose: some preferred physical activity while others preferred to combine exercise and pharmacological treatment. Overall, they were satisfied with taking statins although they preferred not to think about the adverse effects.

### 2nd stage. Identify intervention options

#### Steps 5 and 6. Select intervention functions and specific behavior change techniques

Table 2 summarizes the conducted mapping process, linking practice determinants for inappropriate statin prescription (mainly facilitators) and for providing healthy lifestyle promotion interventions (mainly barriers) to intervention functions and policy categories, ending with potential BCTs for attaining the desired target behavior. For example, the lack of awareness among patients regarding the problem of inappropriate pharmacological prescription (Facilitator of the low-value practice) can be addressed through persuasion (Intervention function) and communication actions (Policy category) enacted by techniques focused on providing information about health consequences (BCT) of this low-value practice.

**Table 2:** Mapping matrix of potential intervention functions, policy categories and Behavior Change Techniques (BCI) to previously identified determinants of inappropriate statin prescription and healthy lifestyle promotion categorized by Theoretical Domains Framework (TDF) dimensions identified from the qualitative study.

**TDF**	**What needs to change (statin** **prescription/healthy lifestyle** **promotion)**	**Intervention function policy** **category**	**Potential BCTs**
Knowledge	Be aware of the problem of inappropriate statin prescription	Education	Feedback on behavior
		Training	Feedback on outcome of the behavior
	Be knowledgeable of the CVD prevention clinical guidelines, especially regarding adequate or recommended care depending on actual CVD risk	Persuasion Enablement	Information about social and health consequences Credible source
	Have updated and unified clinical practice criteria based on independent scientific evidence		Information about others' approval
		*Regulation (principles of practice)*	Social comparison
	*Be aware of the beneficial impact of healthy lifestyles for the prevention of CVD (professionals and patients)*	*Guidelines (mandating changes to adequate service provision) Service provision (training)*	Instruction on how to perform a behavior Demonstration of the behavior
	*Be knowledgeable of the evidence-based healthy lifestyle promotion intervention in primary care (physical activity and healthy diet)*	*Communication/marketing*	Behavioral practice/ rehearsal
			Habit formation
			Behavioral substitution
			Goal setting (behavior)
			Action planning
			Self-monitoring of behavior
			Review behavior goal(s)
			Problem solving
Cognitive and interpersonal skills	Increase skills to estimate and to address/communicate on CVD risk with a focus that goes beyond the numbers and risk factors	Education	Instruction on how to perform a behavior
	Increase skills for appropriate statin prescription	Training	Demonstration of the behavior
		Persuasion	Behavioral practice/rehearsal
	*Have skills in prescribing physical activity and other healthy lifestyles (healthy diet, giving up smoking)*	Enablement	Feedback on behavior
		Environmental restructuring	Review behavior goal(s)
	*Have a standardized protocol that facilitates clinical actions to promote habits*		Self-monitoring of behavior
		*Service provision (continued training/tools)*	Adding object to the environment
		*Guidelines (mandating changes related to service provision)*	Prompts /Cues
		*Communication/marketing*	Goal setting (behavior)
		*Regulation*	Action planning
			Self-monitoring of behavior
Memory, attention, and decision processes	Remember to provide the recommended clinical practice in CVD primary prevention	Training	Prompts/cues
		Environmental restructuring	Framing/reframing
	Remove visual cues that induce an inappropriate approach to high cholesterol in low-risk patients	Enablement	Adding objects to the environment
		*Environmental planning*	Restructuring the physical environment
			Avoidance/reducing exposure to cues for the behavior (inappropriate statin prescription)
Behavioral regulation	Reflect on the performance/practice of inappropriate prescription of statins in primary prevention of CVD	Education Training	Goal setting (behavior) Feedback on behavior
	Have clear and specific objectives, at a personal and organizational level, in reduction of inappropriate statin prescription in primary prevention of CVD	Modeling Enablement	Self-monitoring of behavior
	Have access to data on inappropriate statin prescribing in primary prevention of CVD.	*Service provision (auditing)*	
	*Have access to healthy lifestyles promotion practice data*		
Environmental context and resources	Have a simple tool that favors correct estimation of CVR, according to evidence, that considers additional characteristics of the people (e.g., antecedents)	Environmental restructuring Enablement	Adding/Removing object to the environment Prompts /Cues
	Have support systems in the electronic records that remind about and promote practice in primary prevention of CVD according to the CPGs (avoiding statins and recommending promotion of lifestyles)	Restriction Training	Avoidance/reducing exposure to cues for the behavior Restructuring the physical environment
	Restrict or impede the inappropriate prescription of statins because of simplicity and speed of clinical prescribing conduct		Framing/reframing
		*Guidelines*	Behavior substitution
	*Having tools for a feasible (fast) and effective intervention in healthy lifestyles*	*Service provision (IT support tools in EHR and training)*	Habit formation Associative learning
	*Having access to resources within/outside the health care setting to favor the provision of recommended primary prevention of CVD practice (i.e., healthy lifestyle resources in the community)*		Action planning
			Goal-setting (behavior) (organization level)
	*Nursing participation in the primary prevention of CVD: provision of the recommended intervention to avoid inappropriate prescription*		Demonstration of the behavior Review behavior goals Review outcome goals
Social influences	Patients should be aware of the problem of inappropriate statin prescribing: Risks vs. Benefits	Persuasion Education	Information about social and health consequences Feedback on behavioral outcomes
	Patients must have knowledge of the criteria and practice guidelines: cholesterol, CVD, CVR (patients)	Environmental restructuring Restriction	Credible source Prompts/cues
	The general population must be aware of the problem of excessive medication	Enablement	Framing/reframing Exposure
	The organization must continuously become aware of the problem of inappropriate prescription of statins in healthcare practice (Adaptation; Priority health policies)	*Communication/marketing* *Regulation (organizational priority & standards)*	Review behavior goals Review outcome goals
	The organization must have up-to-date clinical criteria, established in the guidelines based on independent scientific evidence	*Environmental/social planning* *Guidelines*	Discrepancy between current behavior and goal Instructions on how to perform the behavior
	The organization must have a focus beyond the figures and risk factors, both in CPGs and in risk-screening tools and/or interventions	*Legislation*	Action planning Habit reversal
	Advertising or promoting the use of statins in primary prevention of CVD should be restricted		Commitment Removing objects to the environment Avoidance/reducing exposure to cues for the behavior
Professional/social role and identity	Believe that adequate CVD prevention is considered important at their peer and organizational level	Education Persuasion	Information about social and health consequences Feedback on outcomes of the behavior
	Be clear about the criteria for action and responsibilities at the inter-institutional and inter-sectorial level (external: e.g., business medicine) in CVD prevention, based on indication (primary, secondary prevention, etc.)	Modeling Enablement	Credible source Social comparison
	Understand that the role of the doctor goes beyond prescribing drugs		Information about others' approval
		*Communication/marketing*	Identity associated with changed behavior
	Family Medicine and Community Health professionals establishment should be the protagonists (leadership, responsibility) in primary prevention of CVD	*Regulation (organizational priority & standards)*	Valued identity
		*Guidelines (mandating changes to practice and service provision)*	Review behavior goals
	Get other professionals (nurses) involved in the optimization of primary prevention of CVD	*Service provision*	Review outcome goals
			Discrepancy between current behavior and goal
			Instructions on how to perform the behavior
			Action planning
			Habit reversal
			Commitment
Beliefs about consequences	Perceive that not prescribing statins in primary prevention of CVD is not “not treating”	Education Persuasion	Demonstration of the behavior Feedback on outcome(s) of behavior
	Perceive that statins are not more effective than the promotion of habits to avoid CV events in primary prevention of CVD	Modeling	Information about health consequences
		Incentivization	Information about social and environmental consequences
	Perceive that the statin, in primary prevention of CVD, may have adverse effects and is not entirely safe		Credible source
		*Communication/marketing*	Identity associated with changed behavior
	*Have an expectation of the benefits of healthy lifestyle promotion actions (short, medium and long term)*	*Guidelines (evidence diffusion)*	Valued identity
		*Service provision (continued training)*	Information about others' approval
			Social support
			Incompatible beliefs
			Incentive
Beliefs about capabilities	Perceive that one is able and has the necessary skills to provide the healthy lifestyle promotion	Education	Feedback on behavior
		Training	Focus on past success
	Perceive that statin prescribing is not such a simple (low skill) or safe practice	Persuasion	Verbal persuasion about
		Modeling	capability
	Perceive that one is competent and confident enough to carry out the CV risk screening process	Enablement	Vicarious consequences Information about social an environmental consequences
			Information about health consequences
	Perceive that one is competent and confident enough to respond to the sporadic arrival of patients in the target population for CVD primary prevention (they come infrequently), through the promotion of good habits	*Guidelines*	Demonstration of the behavior
		*Service provision (auditing and provision) (continued training)*	Instruction on how to perform a behavior
	Perceive that statin treatment is not so easy for the patient (dosage)		Behavioral practice/ rehearsal
			Credible source
	*Have a sense of self-confidence in prescription of physical activity and other healthy lifestyles*		Problem solving
			Action planning
	*Not have a perception of difficulty in modifying lifestyles (compared to taking a pill)*		Social support (practical)
			Problem solving
Intentions	Should have a strong intention not to prescribe statins inappropriately in primary prevention of CVD	Education	Information about health consequences
		Persuasion	Information about social and environmental consequences
	*Should have a strong intention to provide interventions to promote healthy habits for the primary prevention of CVD*	Incentivization	Credible source
		Modeling	Identity associated with changed behavior
			Discrepancy between current behavior and goal
		*Communication/marketing (evidence diffusion)*	Instructions on how to perform the behavior
		*Regulation (organizational priority & standards)*	Action planning
		*Guidelines (mandating changes to service provision)*	Habit reversal
			Commitment
			Feedback on outcome(s) of behavior
			Incompatible beliefs
			Incentive
			Verbal persuasion about capability
Goals	Have organizational objectives related to the reduction of inappropriate prescription of statins in primary prevention of CVD	Education	Review behavior goals
		Persuasion	Review outcome goals
	Should consider the practice of primary prevention of CVD a priority in accordance with the recommendations.	Incentivization	Discrepancy between current behavior and goal
		Modeling	Instructions on how to perform the behavior
	Should be committed to carrying out a practice of primary prevention of CVD according to the recommendations	Enablement	Goal-setting (behavior)
			Action planning
	*Have the motivation (priority and commitment) to promote lifestyles in primary prevention of CVD*	*Regulation (organizational priority & standards)*	Commitment
		*Guidelines (mandating changes to adequate service provision) Service provision (training)*	Self-monitoring of behavior Monitoring of behavior by others Feedback on behavior Feedback on outcomes of the behavior
Reinforcement	Receive positive or negative reinforcement related to adequate ECV prevention performance	Training Incentivization	Feedback on behavior Material incentive
	Should avoid prescribing out of habit, routine or inertia (to treat cholesterol)	Coercion	(behavior)
		Environmental restructuring	Material reward
			Social reward
		*Service provision (auditing)*	Reward alternative
		*Regulation (principles of practice)*	behavior
			Avoidance/reducing exposure to cues for the behavior (inappropriate statin prescription)
Emotion	Not feel threatened (fear) for not prescribing a drug	Education Persuasion	Feedback on behavior
	Feel confident about not prescribing a statin for CVD primary prevention	Incentivization Coercion	Information about health consequences Credible source
	Experience positive feelings/emotions associated with not doing defensive medicine	*Guidelines*	Discrepancy between current behavior and goal Anticipated regret
	Experience negative emotions when making an inappropriate prescription	*Communication and marketing* *Regulation*	Remove aversive stimulus Information about others' approval
	Feel safe and confident with the action guidelines		Social support

Target behavior: Reduce the prescription of statins in the context of the primary prevention of CVD in low-risk patients and favor the promotion of healthy habits (regular physical activity, healthy diet and giving up smoking) at any opportunistic or programmed office visit for screening or addressing CVD risk factor and/or prevention.

### 3rd stage. Identify implementation procedures

#### Steps 7 and 8. Select strategies, intervention techniques, and modes of execution

Through various round meetings, the working group agreed and drew up a list of 14 potential de-implementation strategies with their respective format and techniques of delivery (Table 3). The specified strategies ranged from the optimization of informatics tools in the EHR used in the routine clinical context of CVD prevention, to update or develop clinical guidelines and educational materials on primary prevention of CVD based in evidence, periodic sending of audit and feedback regarding clinical practice indicators or patient mediated interventions. As an example of BCTs grouping into a potential strategy or intervention component, the editing or updating of a CPG put together at least three identified and selected BCTs that may impact Knowledge: A credible source, gives Instruction on how to perform a behavior, and can guide goal setting related to the behavior (see Table 2).

**Table 3:** Prioritization of the 14 de-implementation strategies derived from the mapping process.

	***Potential effectiveness To what extent do you think this intervention can achieve the desired results in the target population?*** *1: unlikely, 2: unlikely but deserves consideration,* *3: likely, 4: very likely*	***Acceptability*** *To what extent is it acceptable for key agents (PHC professionals, patients, managers, etc.) to use this intervention?* *1: unacceptable, 2: not very acceptable but deserves consideration, 3: acceptable,* *4: highly acceptable*	***Feasibility*** *To what extent do you consider that this intervention can be implemented in the routine clinical context?* *1: unfeasible, 2: unfeasible but deserves consideration, 3: feasible, 4: highly feasible*
New or optimized CVR calculation tool, adding other important risk factors (e.g., family history) to the estimation and/or to help in decision-making	*Very likely*	*Highly acceptable*	*Highly feasible*
Alert and reminder systems (notifications, pop-ups, messages, etc.) in the Medical Record and/or in the prescription system to promote the practice according to the evidence in primary prevention of CVD	*Very likely*	*Highly acceptable*	*Feasible*
Alert and message reminder systems using printed material (e.g., posters, manuals, information sheets, etc.) or interactive means (emails, information capsules, newsletters, etc.) to encourage practice according to the evidence in primary prevention of CVD	*Very likely*	*Acceptable*	*Feasible*
Planning and organization of shared action at health center level, between medicine and nursing, for the provision of clinical intervention in promoting healthy lifestyles	*Very likely*	*Acceptable*	*Feasible*
Formation of a committee of experts to update or develop a corporate guidance document on primary prevention of CVD that includes: a) evidence-based clinical practice recommendations; b) unified criteria for action and responsibilities at the inter-institutional and inter-sectorial level; c) establishment of practice/performance objectives in primary prevention of CVD	*Likely*	*Highly acceptable*	*Feasible*
Elimination of the “asterisk” in blood test results and/or adaptation of the criteria for identification and marking of “case” (e.g., asterisk on cholesterol number >240 mg/dl)	*Likely*	*Acceptable*	*Highly feasible*
Training workshops on primary prevention of CVD and promotion of healthy habits, including training support resources (e.g., clinical intervention manual for promoting healthy habits)	*Likely*	*Acceptable*	*Feasible*
IT tools that facilitate the execution of an intervention to promote lifestyles based on evidence	*Likely*	*Acceptable*	*Feasible*
Corporate campaign “Giving up low-value pharmacological prescribing” promoted by Osakidetza	*Likely*	*Acceptable*	*Feasible*
Tools to aid clinical decision-making in the electronic prescription system, which restrict the inappropriate prescription of statins	*Likely*	*Acceptable*	*Feasible*
Active involvement of the patient in a shared decision-making process in CVD preventive action	*Likely*	*Acceptable*	*Feasible*
Inclusion of practice indicators in primary prevention of CVD in the management and evaluation tools for care performance: a) CV risk registration rate, b) rate of inappropriate prescription of statins in primary prevention in low-risk patients; c) rate of performance of actions to promote lifestyles	*Likely*	*Acceptable*	*Feasible*
Audit/feedback system: Periodic sending of practice or performance indicator reports in inappropriate prescription of statins and actions to promote lifestyles	*Likely*	*Acceptable*	*Feasible*
Edition and publication of educational and informative materials on primary prevention of CVD for patients	*Unlikely*	*Acceptable*	*Feasible*

### Prioritization of de-implementation strategies

Lastly, the potential strategies were sent back to all the healthcare professionals involved in the discussion groups and two health managers for their evaluation regarding three dimensions: acceptability, feasibility and potential effectiveness. Thirteen complete evaluations (13/23) were received that allowed the prioritization of the de-implementation strategies (see Table 3).

## Discussion

This study aimed to report on the application of a systematic, comprehensive, theory-and evidence-informed framework to design potentially effective and feasible de-implementation strategies to favor the abandonment of low-value pharmacological prescribing in CVD primary prevention in low CVD risk patients (17). Specifically, guided by the TDF and the BCW frameworks (13, 23–25), we have conducted a series of actions to identify determinants of low-value practices and behavioral objectives as areas for improvement, which have helped us to design, operationalize and prioritize various de-implementation strategies.

Avoiding or substituting proven potentially harmful, ineffective or inefficient medical practices is important for improving the quality of healthcare while ensuring sustainability of healthcare systems, which is the reason why in recent years the interest in and the evidence base related to successful de-implementation strategies to favor the abandonment of low-value practices has grown quickly. Statins are among the most widely prescribed medications globally and are increasingly used to prevent CVD in people without CVD (“primary prevention”). However, statins have no or low value for the primary prevention of CVD in low-risk patients (18). On the other hand, healthy lifestyle promotion interventions in clinical settings have been shown to be effective and are the preferred recommended practice, especially in low-risk patients (19–21).

From the growing scientific evidence in implementation research it is known that factors determining the implementation of both evidence-based and inappropriate interventions in the clinical setting are multi-level, complex, and context specific (2). Consequently, the design of interventions should be performed following a process of formal analysis of the target behavior and its theoretically predicted mechanisms of action, all guided by models or theories that cover the entire range of possible influences or determinants of the behavior in question (7–10). Through the performed qualitative study with both main involved healthcare professionals (FPs and Practice Nurses) and affected users (low-CVR-risk patients with inappropriate prescription of statins), we have identified multi-level determinants of the target low-value practice within the context of two IHOs in the Basque Health Service-Osakidetza. Almost all of the dimensions of the TDF have been called into play, as at least one practice determinant (barrier or facilitator) was included in these dimensions. Some of the most consistently reported determinants professionals' focus groups were the lack of time and external resources, preferences and characteristics of patients, limitation of available clinical tools and CPGs, social pressures, fears about negative consequences of not treating high cholesterol levels with drugs, and lack of skills and training of professionals in healthy lifestyle promotion. Patients' main determinants were the lack of explanation of the situation at the medical appointment, the desire to be more involved in the treatment decision, belief and concern about family health history in the decision, and the lack of healthcare professional's recommendation about healthy lifestyles with or without prescription of statins.

The identified determinants are in line with other determinants identified or reported in previous studies regarding determinants of low-value practice and of low-value pharmacological prescription. For example, uncertainty due to the variability and/or conflict of the guidelines with respect to the recommended practice, the pressures and demands on the part of the patients, the need for rapid and decisive action in response to the reasons for consultation and the desire to please the patients have been identified as interconnected motives that generally justify maintaining low-value practices (27). With regard to inappropriate prescription of drugs in general, a systematic review published by Anderson et al. (28), in which the barriers and facilitators for inappropriate prescription were explored, highlights four aspects that facilitate or hinder professionals' decisions when faced with a possible pharmacological prescription: first, awareness of the problem, i.e., knowing to what extent the clinical practice of each professional conforms to what is recommended in CPGs, as well as knowing the consequences of treating a patient pharmacologically or not. Second, self-efficacy, which encompasses the professional's ability to manage the clinical situation based on their knowledge or their ability to offer a non-pharmacological alternative, among others. The third aspect to highlight is inertia, which is a barrier to change in clinical practice; and finally, feasibility, where all the external factors that affect the clinical decision would be included: patient characteristics and preferences, social/cultural factors, prescriptions made by another professional, group pressure and so on. Studies carried out exclusively on the inappropriate prescription of statins emphasized the influence of the perception that professionals have of each patient's CVR and their opinion about the effectiveness and safety of statins (29), beliefs or attitudes toward behavior and perceived control (30); the additional risk factors that the patient may present and the patient's preferences about receiving drug treatment or not (31).

One peculiar aspect in this point is that, due to the addressed clinical scenario (the reduction of low-value prescribing of statins in CVD primary prevention where the promotion of healthy lifestyles is the alternative, recommended practice), this project has attempted to simultaneously identify determinants of both clinical practices. Although it may seem obvious, in such clinical scenarios, stress must be placed on identifying the factors that facilitate or maintain the low-value practice, and on the other hand, the barriers that impede the recommended practice (32).

With the main goal of designing and developing targeted strategies that address the specific determinants of CVD prevention practice in the Basque Health Service-Osakidetza, the present's study main action has been to carry out a mapping process of de-implementation and implementation strategies in order to reduce low-value practices (inappropriate statin prescribing) and promote the implementation of the recommended practice (healthy lifestyle interventions), based on the determinants of routine practice reported by FPs in the focus groups, following the procedure established by the BCW.

The 14 strategies that have emerged from the mapping processes are all “old known” strategies and interventions. Nevertheless, previous studies targeting the reduction of low-value statin prescription have shown some effectiveness of certain dissemination strategies as informative web pages or the implementation of electronic CPGs when compared to routine practice especially when used as multi-component strategies (33–37). Further, educational or training actions for professionals (webinars and workshops), have also shown some effectiveness, especially when combined with other interventions in multi-component strategies (33, 34). And lastly, audit and feedback interventions or those sending a clinical case scenario to professionals (38), and techniques to aid decision-making through clinical decision support systems have achieved good results in increasing the registering of CVR and in adjusting the prescription (39–41).

However, the innovative contribution of having used the BCW is that, both actions, determinant identification and mapping of strategies, aim to target the specific clinical behavior most likely to enable the desired change prioritized by the research group and professionals involved: physicians' decision-making regarding the therapeutic option. Moreover, following a taxonomy of choice architecture techniques (42), all except one of the 14 identified strategies may be categorized as influencing FPs decision-making through three different modes: decision information (e.g., dissemination of CPGs), decision assistance (i.e., alert and reminder systems; involvement of the patient in a shared decision-making process), and decision structure (e.g., audit and feedback system). Furthermore, the agents involved have prioritized the resulting potential de-implementation strategies after assessing their perceived acceptability, feasibility and potential effectiveness. Therefore, though not innovative interventions or strategies, those identified are those that address the specific determinants identified by the protagonists. Research is now needed however, to test whether these barrier-specific strategies for de-implementation identified in the present study are also effective in our context (17).

The present study has several limitations. First, the formative study has been performed in only two IHO of Basque Health Service-Osakidetza that are not representative of all Primary Care centers within our health service. Second, after having invited all professional within the two participating IHOs, only a reduced and auto-selected sample was obtained and we cannot guarantee that we have reached saturation of data regarding physicians discourse related to inappropriate statin prescription. And finally, this previous issue also extends to patients groups by limiting the planned triangulation of discourses to only one group of patients.

## Conclusion

The present study aims to contribute to the body of currently scarce literature available on practical de-implementation initiatives by providing detailed illustrations/explanations of our stepped, systematic approach to the design and development of targeted behavior change actions based on prominent available frameworks and theories, mostly from implementation science. Key research questions in implementation science also involve determining what implementation strategies should be provided, to whom, and when, to achieve optimal success in implementing evidence-based clinical practice. As the same paradigm must apply for de-implementation of low-value practices, we propose now to investigate the comparative effectiveness of some/different types or intensities of the prioritized strategies in Phase II of the DE-imFAR project. The future evaluative phase of our study will have the aim of increasing evidence on whether the specific strategies that address determinants of recommended practice in CVD prevention, some similar to those evaluated in the few studies conducted to date, are also effective in our context. If the strategies explored are successful, health planners and managers will have the evidence needed to support the introduction of such structured strategies, informed by the application of methods and procedures of the emerging science of implementation and de-implementation.

## Data availability statement

The original contributions presented in the study are included in the article/Supplementary material, further inquiries can be directed to the corresponding author.

## Ethics statement

The studies involving human participants were reviewed and approved by Basque Country Clinical Research Ethics Committee (Reference: PI2019102, approved on 10/04/2019). The patients/participants provided their written informed consent to participate in this study.

## Author contributions

AS, JP, and GG conceived the idea, the study guarantors, primarily responsible for the study design and planning, as well as the funding obtained, and will be responsible for project coordination and supervision, analysis and interpretation of results, and manuscript preparation. SP, UE-A, SG-L, MM, IL, RS, ML, OL, and CH collaborated in the study design and were responsible for study coordination, interpretation of results, and manuscript preparation. AS, UE-A, and MM were responsible of the performed analysis. All authors critically reviewed the manuscript and approved this version submitted for publication.

## Funding

This project was supported by the Basque Government Department of Health (funded project 2018111085 and 2021111024) and by the Carlos III Institute of Health and co-funded by the European Union (funded project Grant Nos. PI21/00025, RD16/0007/0002, and RD21/0016/0003). The funding bodies had no role in the design of the study, collection, analysis, and interpretation of data or the writing of the manuscript.

## Conflict of interest

The authors declare that the research was conducted in the absence of any commercial or financial relationships that could be construed as a potential conflict of interest.

## Publisher's note

All claims expressed in this article are solely those of the authors and do not necessarily represent those of their affiliated organizations, or those of the publisher, the editors and the reviewers. Any product that may be evaluated in this article, or claim that may be made by its manufacturer, is not guaranteed or endorsed by the publisher.
