# Supplementary material for: Applying the behavior change wheel to design de-implementation strategies to reduce low-value statin prescription in primary prevention of cardiovascular disease in primary care
Source: Front Med (Lausanne). 2022 Oct 13;9:967887. doi: 10.3389/fmed.2022.967887 (PMC9606599; doi:10.3389/fmed.2022.967887)
Supplement: Supplementary file 1 [file Table_1.pdf]

S1 Table 1. Target behavior specification (BCW's Worksheet 3)

**Who needs to perform the behavior?**	Family physicians and nurses
**What do they need to do differently to achieve the desired change?**	Provide the recommended healthy lifestyle promotion intervention, instead of prescribing statins, for the primary prevention of CVD in low-risk patients
**When do they need to do it?**	In opportunistic or programmed screening/management of CVD risk factors
**Where do they need to do it?**	Opportunistic or programmed consultations/visits
**How often do they need to do it?**	At any encounter feasible
**With whom do they need to do it?**	Patients with low CVD risk (<10) aged ≥40 years old in men and ≥45 years old in women
**Target behavior**	*Reduce the prescription of statins in the context of the primary prevention of CVD in low-risk patients (REGICOR <5%) and favor the adoption and implementation of the recommended intervention, the promotion of healthy habits (regular physical activity, healthy diet and cessation of smoking) at any opportunistic or programmed screening or addressing of CVD risk factors in health center visits.*

S1 Table 2. List of candidate target behaviors (BCW's Worksheet 2-Task1)

Precipitating factors for CVD primary prevention practice

- alarm system (PAPPs) or
- in the case of high cholesterol level (analytical results)
- presence of a prescription initiated or suggested by another professional (specialist or private)

Steps / actions involved in the primary prevention of CVD

Introduce/frame the clinical scenario or episode to the patient in the context of the primary prevention of CVD, demystifying the relevance of an elevated cholesterol level

Ensure diagnosis by requesting the test for its repetition

Communicate the clinical scenario to the patient based on a confirmed value (e.g., cholesterol ≥220 mg/dl), including obtaining additional clinical information related to other risk factors (diabetes, blood pressure, family history, etc.)

Calculate CV risk in the tool for this purpose in the Clinical Information System. In the case of absence of parameters or outdated information for the calculation of the CVR, proceed to or request the necessary tests/analytics

Consider possible treatment actions (drug treatment; promotion of healthy habits) based on:

- Estimated risk and clinical parameters (e.g., cholesterol level)
- Knowledge, heuristics, attitudes, health professional's skills
- Characteristics of the specific patient
- Other contextual factors (time, overload, decisional fatigue, organizational norms, external pressure, etc.)

Initiate a shared decision-making process in which you discuss intervention options, provide benefit and risk information, propose best practice, and collect patient feedback.

Jointly select the promotion of healthy habits as a preferred preventive action

Provide an intervention to promote healthy habits, through verbal advice, relying on documentation, materials or support resources (e.g., CV risk calculator)
